# Cost-Effectiveness Analysis of Xiyanping Injection (Andrographolide Sulfonate) for Treatment of Adult Community Acquired Pneumonia: A Retrospective, Propensity Score-Matched Cohort Study

**DOI:** 10.1155/2019/4510591

**Published:** 2019-03-18

**Authors:** Honghao Shi, Wanjie Guo, He Zhu, Meng Li, Carolina Oi Lam Ung, Hao Hu, Sheng Han

**Affiliations:** ^1^State Key Laboratory of Quality Research in Chinese Medicine, Institute of Chinese Medical Sciences, University of Macau, Macau; ^2^International Research Center of Medical Administration, Peking University, Beijing, China

## Abstract

Xiyanping injection (andrographolide sulfonate) has shown clinical effects on community acquired pneumonia. However, there is little known about the effectiveness and costs of combining Xiyanping injection with conventional treatment on adult community acquired pneumonia in daily practice. The aim of this study was to evaluate the cost-effectiveness of combining Xiyanping injection with conventional treatment for treatment of adult community acquired pneumonia by comparing with conventional treatment from a societal perspective. Using retrospective cohort method, this study demonstrates that Xiyanping injection combined with conventional treatment is superior to conventional treatment for patients using cephalosporins and antibiotics under the effectiveness index of length of hospital stay and is more cost-effective.

## 1. Introduction

Community acquired pneumonia (CAP) refers to inflammation of the infectious pulmonary parenchyma, including the pneumonia caused by pathogens with definite latency after admission. The annual incidence of CAP is 1-12%; in particular, the morbidity of adult CAP is 5~11/1000 persons/year in Europe and North America and 2.5/1000 persons/year in the United States [1]. Japanese studies show that the incidences of CAP in 15-64 years old, 65-74 years old, and 75+ years old are 3.4/1000 persons/year, 10.7/1000 persons/year and 42.9/1000 persons/year, respectively [2]. In 2016, a retrospective study was conducted in Zhuhai City, China. It was shown that the percentage of male CAP patients was higher than female ones; most patients were under 5 years old or over 60 years old, and autumn and winter were the main onset seasons [3].

Consequently, CAP has caused a heavy economic burden on patients and the health care systems worldwide [4–8]. According to the Expert Consensus on the Treatment of Community Acquired Pneumonia (2014 edition), the treatment of CAP in China can be divided into traditional Chinese medicine (TCM) treatment and western medicine treatment. Among the TCM syndrome differentiation and treatment for CAP, Xiyanping injection is a kind of common medicine for phlegm-heat obstructing the lung (one disease name in TCM sharing a similar syndrome with CAP). The main component of this injection is sulfonated andrographolide. Modern pharmacological studies show that andrographolide has effects of antipyretic, anti-inflammatory, and antiviral. Xiyanping is obtained from the main component of Chinese herbal medicine andrographolide by sulfonation process. The main components are andrographolide total lactone, dehydroandrographolide, and andrographis. It has anti-inflammatory, antipyretic, antiviral, and immune regulation and other pharmacological effects, clinically used for the treatment of upper respiratory tract infections, viral pneumonia, bronchitis, diarrhea in children, bacillary dysentery, and acute heat diseases. Xiyanping injection was produced exclusively by Jiangxi Qingfeng Pharmaceutical Co., Ltd., which entered Catalogue B of the National Drugs Catalogue of Basic Medical Insurance, Industrial Injury Insurance and Reproductive Insurance (2017 edition). It is limited to severe patients in secondary and higher medical institutions.

There are some clinical studies on Xiyanping injection in the treatment of CAP in China, where the control group is antibiotics and the experimental group is antibiotics combined with Xiyanping injection. Shu et al. conducted a comparative study in 2013, which included 60 CAP patients in both Xiyanping injection group and conventional treatment group, the outcome including relieving fever, cough, sputum disappearance time, and adverse reactions, whose results showed the Xiyanping injection had better effects [9]. A study in 2014 involved 140 CAP in Xiyanping injection group and 140 CAP patients in antibiotic group, which set the rate of fever and length of hospital stay as outcome indicators, and the Xiyanping injection had better effects [10]. In 2017, Deng et al. found Xiyanping injection group had a better effect in reducing length of hospital stay, incidence of adverse reactions, and clinical indicators (onset and disappearance of fever and cough, C-reactive protein (CRP) returned to normal, and lung shadow absorption time) compared with Azithromycin group in a comparative experimental study [11].

Xiyanping injection showed better effects in these studies. At present, the current pharmacoeconomic evaluation of Xiyanping injection mainly focuses on children's pneumonia, children's upper respiratory tract infection, enteritis, and hand-foot-and-mouth disease [12–14]. However, there is no pharmacoeconomic evaluation of Xiyanping injection for adult CAP treatment in China.

Therefore, this study explored the differences in cost and health output between Xiyanping injection exposure group and nonexposure group for CAP patients with different patterns of drug use in the real world and conducted pharmacoeconomic evaluation in order to provide references for clinicians to optimize treatment options and for health insurance departments to make relevant policies.

## 2. Method

### 2.1. Research Design

**Figure 1:**
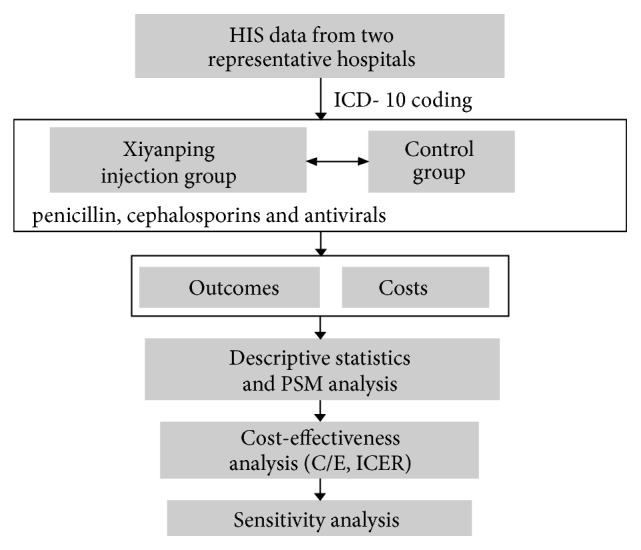
Flowchart of research process.

This study was a retrospective cohort study to compare the cost and outcome of conventional treatment combined with Xiyanping injection and conventional treatment for hospitalization patients diagnosed as adult community acquired pneumonia. The whole research flowchart is depicted (see Figure 1).

### 2.2. Research Materials

In this study, data was extracted from the Health Information System (HIS) of two hospitals, including Ganxian People's Hospital (Grade II class A) and Jiangxi Traditional Chinese Medicine Hospital (Grade III class A). On the basis of ICD-10 coding, this study included J13-15 and J18 and excluded patients whose case information was incorrect or incomplete, where the year of extraction was from 2013 to 2015.

The included patients were divided into three subsamples following their patterns of drug use: penicillin, cephalosporin, and antiviral. Then, in each subsample, the patients were divided into Xiyanping combination group (Xiyanping injection combined with conventional treatment) and control group (conventional treatment only).

### 2.3. Intervention

According to the Guideline for the Diagnosis and Treatment of Adult CAP in China, doctors need to analyze the etiology of patients according to their age, underlying diseases, severity, and past medication history and then select appropriate anti-infective drugs and medication regimens after patients are diagnosed as CAP [15]. Pneumonia can be simply divided into bacterial pneumonia and viral pneumonia according to different pathogens. At present, antibiotics and antiviral drugs are mainly used to treat CAP, where nearly 80% of antibiotics are penicillin and cephalosporins being beta-lactam antibiotics [16]. Thus, following the main anti-infective drugs used in clinic, in this study, three types of conventional treatment were studied: penicillin, cephalosporin, and antiviral. Then Xiyanping combination group and control group of each conventional treatment were compared, respectively:

- Xiyanping combination group versus penicillin group.
- Xiyanping combination group versus cephalosporin group.
- Xiyanping combination group versus antiviral group.

### 2.4. Study Outcomes

The outcome indicators of this study included the length of hospital stay, laboratory examination indicators, and treatment effects. Among them laboratory examination indicators include C-reactive protein (CRP), white blood cell count, neutrophil count, and percentage of neutrophils. CRP is a sensitive acute reactive protein synthesized by the liver, where its concentrations are very low in the serum of healthy people and will increase significantly when bacterial infection or tissue injury occurs. Therefore, CRP level can be used as one index for inflammation. In addition, white blood cell count and neutrophil percentage are also important detection indicators for the observation of various infections [17–19]. All these indicators have important implications for the selection of clinical interventions to reduce infection. In this study, the neutrophil proportion, neutrophil count, and white blood cell count were used to measure the effects of infections, which also represent the clinical effects. In the results, the outcome of CRP, neutrophil proportion, neutrophil count, and white blood cell count were reflected as the difference value (Mean±SD) between preadmission and postdischarge (admission laboratory values-discharge laboratory values). Therapeutic effects are divided into cure, improvement, unhealed, death, unknown, and others.

### 2.5. Costs

In this study, the cost only considered the direct medical cost in the period of 2013–2015, including total hospitalization costs, medicine fees, laboratory fees, bed fees, operation fees, nursing fees, inspection fees, treatment fees, and other expenses. Among them, the cost of medicine includes traditional Chinese medicine and western medicine. Cost data come from HIS system of Ganxian People's Hospital and Jiangxi Traditional Chinese Medicine Hospital.

### 2.6. Propensity Score Matching

There are many confounding variables in this study, so Propensity Score Matching (PSM) is used to control the confounding factors and reduce the impact of confounding factors on the evaluation of intervention effect. The research steps of PSM include confirming the selection of PSM, estimating propensity score, choosing propensity score method, conducting equilibrium test, estimating processing effect, and conducting sensitivity analysis. In this study, logistic regression was used to score the tendency, mixed variables including social demographic characteristics, pretreatment information, treatment conditions (age, sex, occupation, marital status, ICU, CRP, neutrophil count, percentage of neutrophils, white blood cell count, and comorbidities). PSM was achieved by Nearest Neighbor Matching (NNM) in the matchit bag of R language, where the matching ratio is set to be 1:1.

### 2.7. Estimated Differences and Cost-Effectiveness Analysis

In this study, descriptive statistics and PSM analysis were carried out in three aspects: description of basic characteristics, comparative analysis of outcome, and cost. The outcome, hospitalization cost and composition, percentage, and P-value of the two groups were also compared and analyzed by SPSS statistical software and matchit package in R language; P < 0.05 was considered statistically significant. In the cost-effectiveness analysis, the cost data and outcome indicators are derived from the results of PSM matching. Then two commonly used metrices including cost/effectiveness (C/E) and incremental cost-effectiveness ratio (ICER) are adopted to determine which group would bring more economic benefit to CAP patients.

### 2.8. Sensitivity Analysis

Variables in pharmacoeconomic research are usually uncertain, and some factors would influence the results. To verify the impact of changes in some factors, data uncertainty should be analyzed. We assumed the patient's drug cost up or down 20% to observe the stability of cost-effectiveness analysis.

## 3. Results

### 3.1. Sample Characteristics at Baseline before PSM

#### 3.1.1. Sample Characteristics at Baseline before PSM: Xiyanping Combination versus Penicillin

As shown in Table 1, at baseline, there were significant difference between Xiyanping combination group and penicillin group in terms of age, gender, marriage, and occupation.

#### 3.1.2. Sample Characteristics at Baseline before PSM: Xiyanping Combination versus Cephalosporin

Before PSM, at baseline, 186 patients were included into Xiyanping combination group, while 2,791 patients were included into cephalosporin group. Between the two groups, there were significant differences in age, marriage, occupation, CRP, neutrophil proportion, neutrophil count, and white blood cell count (see Table 2).

#### 3.1.3. Sample Characteristics at Baseline before PSM: Xiyanping Combination versus Antiviral

At baseline, before PSM, 65 patients were included into Xiyanping combination group, while 228 patients were included into antiviral group. There were significant differences in age, occupation, and neutrophil proportion between the two groups (see Table 3).

### 3.2. Sample Characteristics at Baseline after PSM

#### 3.2.1. Sample Characteristics at Baseline after PSM: Xiyanping Combination versus Penicillin

After PSM, at baseline, as shown in Table 4, there was no difference at baseline between the Xiyanping combination group and penicillin group (see Table 4). In each group, 139 patients were finally included.

#### 3.2.2. Sample Characteristics at Baseline after PSM: Xiyanping Combination versus Cephalosporin

After PSM, as shown in Table 5, there was no significant difference between Xiyanping combination group and cephalosporin group. In each group, 180 patients were finally included.

#### 3.2.3. Sample Characteristics at Baseline after PSM: Xiyanping Combination versus Antiviral

After PSM, at baseline there was no significant difference between the two groups (see Table 6). In each group, 43 patients were finally included.

### 3.3. Outcomes

#### 3.3.1. Outcomes: Xiyanping Combination versus Penicillin

As shown in Table 7, in terms of hospitalization stay CRP difference, neutrophil count difference, white blood cell count difference, and cure effect, no significant difference was found between Xiyanping combination treatment and penicillin treatment, except neutrophil proportion difference.

#### 3.3.2. Outcomes: Xiyanping Combination versus Cephalosporin

Compared with cephalosporin treatment, Xiyanping combination treatment had less hospitalization stay (7.84±3.31 versus 8.47±3.29;* P* = 0.070) and higher neutrophil proportion difference (20.40±18.60 versus 6.82±11.64;* P* = 0.006) (see Table 8). However, there was no any statistical significance in other outcome measures.

#### 3.3.3. Outcomes: Xiyanping Combination versus Antiviral

Between Xiyanping combination treatment and antiviral treatment, for hospitalization stay, CRP difference, and cure effect, there was no significant difference (see Table 9). But significant differences existed in neutrophil proportion difference, neutrophil count difference, and white blood cell count difference.

### 3.4. Costs

#### 3.4.1. Costs: Xiyanping Combination versus Penicillin

The total hospitalization cost of Xiyanping combination treatment (10,506.10±7,722.98) was higher than that of penicillin treatment (9,573.51±6,113.09), but without statistical significance (*P* = 0.266) (see Table 10). Also, there was no statistical difference in subcost items between the two treatment groups.

#### 3.4.2. Costs: Xiyanping Combination versus Cephalosporin

For the cost comparison between Xiyanping combination treatment and cephalosporin treatment, there was no statistically significant difference for total hospitalization cost (see Table 11). But the mean of TCM fee in Xiyanping treatment group (885.20 CNY) was significantly higher than that in cephalosporin treatment group (676.27). The mean of bed fee and examination fee in Xiyanping combination treatment group was significantly lower than those of cephalosporin treatment group.

#### 3.4.3. Costs: Xiyanping Combination versus Antiviral

For the cost comparison between Xiyanping combination treatment and antiviral treatment, there was no statistically significant difference in total hospitalization cost (see Table 12). But the bed fee, examination fee, and other fee of cephalosporin treatment were significantly higher than those of Xiyanping combination treatment.

### 3.5. Results of CEA

#### 3.5.1. CEA: Xiyanping Combination versus Penicillin

As shown in Table 13, regarding hospitalization stay, Xiyanping combination treatment was associated with a shorter hospitalization stay (-0.36 days) compared with penicillin treatment, but with an additional cost (932.00 CNY). The ICER for Xiyanping combination treatment was 2,589.89 CNY/day compared with penicillin treatment, meaning that reducing one day of hospitalization stay needs extra 2,589 CNY.

Regarding cure rate, compared with penicillin treatment, Xiyanping combination treatment was associated with a higher cost (932.00 CNY) but with a lower cure rate (-4.30%), showing a cost-effectiveness disadvantage for Xiyanping combination treatment.

#### 3.5.2. CEA: Xiyanping Combination versus Cephalosporin

As shown in Table 14, in comparison to cephalosporin treatment Xiyanping combination treatment was associated with a shorter hospitalization stay (-0.63 days) while being with a lower cost (352.00 CNY). This indicates that Xiyanping combination treatment demonstrates a dominated cost-effectiveness advantage.

In terms of cure rate, the ICER for Xiyanping combination treatment was 320.00 CNY/percentage compared with cephalosporin treatment, meaning that increasing one percentage of cure rate needs extra 320.00 CNY.

#### 3.5.3. CEA: Xiyanping Combination versus Antiviral

As shown in Table 15, compared with antiviral treatment, Xiyanping combination treatment was associated with a shorter hospitalization stay (-1.05 days) and a lower cost (-4,572.00 CNY), indicating a dominated cost-effectiveness.

Regarding cure rate, Xiyanping injection treatment was associated with a lower cure rate (11.60%) compared with antiviral, but with a lower cost (-4,572.00 CNY). The ICER of Xiyanping combination treatment was 394.14 CNY/percentage, meaning that increasing one percentage of cure rate needs extra 394.14 CNY.

## 4. Discussion

From the cost and outcomes, there was no significant difference between conventional treatment and conventional treatment combined with Xiyanping injection in penicillin group, cephalosporin group, and antiviral group. From the results of pharmacoeconomic, when the length of hospital stay is the effect indicator, there is no difference in cost-effectiveness between the two groups in statistical significance. In terms of absolute value, in the cephalosporin group and the antiviral drug group, Xiyanping injection combined with conventional treatment is more economical than conventional treatment only. When the clinical cure rate is used as the effect indicator, there is no statistical difference between the two groups. In terms of absolute value, Xiyanping injection combined with conventional treatment had no economic benefit in penicillin patients. The incremental cost-effectiveness ratio in the other two groups needed to be compared with the threshold to determine whether it had economic benefit or not. Threshold is the willingness to pay determined by doctors and patients together.

For Xiyanping injection treatment, it also has some adverse reactions occasionally, such as rash, itching, fever, chills, pain, irritability, rare shortness of breath, cyanosis, palpitations, convulsions, etc. Zeng et al. reviewed 27 cases of adverse reactions caused by Xiyanping injection. They found the adverse reactions caused by Xiyanping injection were not related to gender and occurred mostly in the <10-year-old age group, and the appearance time occurred mostly within the first 30 minutes after injection. The main clinical manifestations of adverse reactions are allergies and intestinal fistula [20]. A meta-analysis of distribution characteristics of adverse reactions of Xiyanping injection was conducted in 2018, in which a total of 1578 studies were included. It concluded the adverse reactions rate (ADR) was 1.8% (95% confidence interval: 1.7% to 2.1%), and the higher ADR was related to higher frequency, longer time of injection, and the combination with other drugs [21]. In 2018, an observation study on adverse reactions of Xiyanping injection found that, compared with the drug dose and the combination of drugs, the age-related adverse reactions were more obvious (*P* < 0.05); compared with the nervous system, digestive system, and respiratory system, the skin was the main organ involved in adverse reactions (*P* < 0.05), so it is necessary to strengthen the rational use of Xiyanping injection [22]. However, most of the adverse reactions will return to normal after stopping the drug. In addition, it is contraindicated for allergic people, pregnant women, and children under one year. Therefore, practitioners also need to pay special attention to clinical use of Xiyanping injection.

In addition, there are some limitations of this research which needs to be addressed. First, in this study the dosage of different treatment has not been standardized because we used the real world data of electronic health record data that just recorded the realistic utilization data. Future study can use more big data panel to investigate the dosage standards. Second, this study focused on adult CAP. Since Xiyanping injection was also applied to children and aged person, future study should investigate the cost-effectiveness of Xiyanping injection on CAP of those population.

## 5. Conclusion

This study conducts the cost-effective analysis of Xiyanping injection combined with conventional treatments and conventional treatments (control group) for patients with CAP, where three representative conventional treatments are selected including penicillin, cephalosporin, and antiviral. It is concluded that when hospitalization stay is chosen as the effect index, Xiyanping injection is more economically efficient than conventional treatments in cephalosporin and antiviral groups; the efficiency in penicillin group should be further evaluated by comparing ICER value with a threshold and common willingness to pay determined by doctor and patient. While when cure rate is chosen as the effect index, Xiyanping injection is not economically efficient in penicillin group, the efficiency in cephalosporin and antiviral groups should be further evaluated similarly. Therefore, clinicians could prescribe specific patients with Xiyanping injection combined with conventional treatment drugs. Health care system can also refer to this study, adjust or formulate policies to reduce the disease burden of patients with CAP, improve their quality of life, and improve the level of comprehensive prevention and treatment of CAP as well.

## Figures and Tables

**Table 1:** Characteristics at baseline before PSM: Xiyanping combination versus penicillin.

Variable	Xiyanping combination		Penicillin		*P*
	N	%	N	%	
*Sample size*	143	6.2%	2174	93.8%	
*Age (y), mean ± SD*	58.64±18.77		64.46±16.31		<0.001
*Gender*					
Male	75	52.4%	1137	52.3%	0.025
Female	68	47.6%	1037	47.7%	0.025
*Marriage*					
Widowed	6	4.2%	54	2.5%	0.074
Married	117	81.8%	1848	85.2%	0.001
Single	20	14.0%	263	12.1%	0.095
Divorced or others	0	0.0%	5	0.2%	0.607
*Occupation*					
Peasants	18	15.5%	1026	50.9%	<0.001
Employment	61	52.6%	586	29.1%	<0.001
Unemployment	0	0.0%	10	0.5%	0.447
Retirement	1	0.9%	128	6.3%	0.016
Unknown	33	28.4%	195	9.7%	<0.001
Others	3	2.6%	71	3.5%	0.592
*Comorbidities*					
Yes	3	2.1%	17	0.8%	0.099
No	140	97.9%	2157	99.2%	0.099
*CRP difference*	51.14±52.04		44.41±57.95		0.529
*Neutrophil proportion difference*	78.52±11.91		73.90±13.11		0.067
*Neutrophil count difference*	7.12±2.59		6.79±4.42		0.535
*White blood cell count difference*	9.04±2.80		8.78±5.16		0.656

**Table 2:** Characteristics at baseline before PSM: Xiyanping combination versus cephalosporin.

Variable	Xiyanping combination		Cephalosporin		*P*
	N	%	N	%	
*Sample size*	186	6.2%	2791	93.8%	
*Age (y), mean ± SD*	57.10±19.52		64.56±16.10		<0.001
*Gender*					
Male	95	51.1%	1405	50.3%	0.846
Female	91	48.9%	1386	49.7%	0.846
*Marriage*					
Widowed	3	1.6%	70	2.5%	0.442
Married	147	79.0%	2444	87.8%	0.001
Single	35	18.8%	266	9.6%	<0.001
Divorced or others	1	0.5%	4	0.1%	0.204
*Occupation*					
Peasants	42	25.8%	1491	56.7%	<0.001
Employment	71	43.6%	601	22.9%	<0.001
Unemployment	0	0.0%	15	0.6%	0.333
Retirement	7	4.3%	180	6.8%	0.206
Unknown	35	21.5%	233	8.9%	<0.001
Others	8	4.9%	108	4.1%	0.620
*Comorbidities*					
Yes	8	4.3%	13	0.5%	<0.001
No	178	95.7%	2778	99.5%	<0.001
*CRP difference*	60.51±51.43		38.77±54.65		0.002
*Neutrophil proportion difference*	79.82±13.51		73.60±13.43		0.001
*Neutrophil count difference*	8.06±4.73		6.62±4.76		0.027
*White blood cell count difference*	9.80±4.92		8.99±11.46		0.258

**Table 3:** Characteristics at baseline before PSM: Xiyanping combination versus antiviral.

Variable	Xiyanping combination		Antiviral		*P*
	N	%	N	%	
*Sample size*	65	22.2%	228	77.8%	
*Age (y), mean ± SD*	46.14±19.71		59.47±18.49		<0.001
*Gender*					
Male	32	49.2%	108	47.4%	0.791
Female	33	50.8%	120	52.6%	0.791
*Marriage*					
Widowed	0	0.0%	2	0.9%	0.450
Married	56	87.5%	211	93.4%	0.125
Single	8	12.5%	11	4.9%	0.029
Divorced or others	0	0.0%	2	0.9%	0.450
*Occupation*					
Peasants	37	57.8%	155	68.0%	0.130
Employment	8	12.5%	22	9.6%	0.507
Unemployment	1	1.6%	5	2.2%	0.753
Retirement	3	4.7%	17	7.5%	0.438
Unknown	9	14.1%	8	3.5%	0.001
Others	6	9.4%	21	9.2%	0.968
*Comorbidities*					
Yes	4	1.8%	9	13.8%	0.446
No	61	93.8%	219	336.9%	0.446
*CRP difference*	39.39±43.63		35.41±49.32		0.560
*Neutrophil proportion difference*	76.74±14.66		72.33±11.90		0.049
*Neutrophil count difference*	6.14±2.85		5.93±3.84		0.672
*White blood cell count difference*	7.88±3.12		7.88±4.00		0.996

**Table 4:** Characteristics at baseline after PSM: Xiyanping combination versus penicillin.

Variable	Xiyanping combination		Penicillin		*P*
	N	%	N	%	
*Sample size*	139		139		
*Age (y), mean ± SD*	58.45±18.54		59.12±16.91		0.753
*Gender*					
Male	74	53.2%	68	48.9%	0.472
Female	65	46.8%	71	51.1%	0.472
*Marriage*					
Widowed	113	81.3%	108	77.7%	0.458
Married	20	14.4%	24	17.3%	0.511
Single	6	4.3%	7	5.0%	0.776
Divorced or others	0	0.0%	0	0.0%	0
*Occupation*					
Peasants	3	2.7%	1	0.9%	0.326
Employment	18	16.1%	17	15.6%	0.326
Unemployment	59	52.7%	55	50.5%	0.741
Retirement	0	0.0%	0	0.0%	1
Unknown	31	27.7%	35	32.1%	0.472
Others	1	0.9%	1	0.9%	0.985
*Comorbidities*					
Yes	3	2.2%	2	1.4%	0.652
No	136	97.8%	137	98.6%	0.652
*CRP difference*	47.64±50.05		60.42±67.77		0.455
*Neutrophil proportion difference*	77.47±11.80		75.57±9.91		0.546
*Neutrophil count difference*	7.11±2.69		5.94±2.65		0.130
*White blood cell count difference*	9.14±2.90		10.84±16.65		0.600

**Table 5:** Characteristics at baseline after PSM: Xiyanping combination versus cephalosporin.

Variable	Xiyanping combination		Cephalosporin		*P*
	N	%	N	%	
*Sample size*	180		180		
*Age (y), mean ± SD*	57.33±19.06		58.95±19.09		0.578
*Gender*					
Male	91	50.6%	87	48.3%	0.673
Female	89	49.4%	93	51.7%	0.673
*Marriage*					
Widowed	144	80.0%	140	77.8%	0.605
Married	32	17.8%	31	17.2%	0.890
Single	3	1.7%	8	4.4%	0.126
Divorced or others	1	0.6%	1	0.6%	1
*Occupation*					
Peasants	8	5.1%	8	5.2%	0.968
Employment	41	26.1%	34	22.1%	0.405
Unemployment	70	44.6%	72	46.8%	0.701
Retirement	0	0.0%	0	0.0%	1
Unknown	32	20.4%	36	23.4%	0.523
Others	6	3.8%	4	2.6%	0.541
*Comorbidities*					
Yes	5	2.8%	1	0.6%	0.100
No	175	97.2%	179	99.4%	0.100
*CRP difference*	59.56±50.62		72.46±70.55		0.290
*Neutrophil proportion difference*	79.71±13.75		79.31±10.98		0.860
*Neutrophil count difference*	8.07±4.81		7.32±4.16		0.380
*White blood cell count difference*	9.81±5.01		8.98±4.38		0.350

**Table 6:** Characteristics at baseline after PSM: Xiyanping combination versus antiviral.

Variable	Xiyanping combination		Antiviral		*P*
	N	%	N	%	
*Sample size*	43		43		
*Age (y), mean ± SD*	52.21±19.54		54.70±21.70		0.578
*Gender*					
Male	21	48.8%	23	53.5%	0.666
Female	22	51.2%	20	46.5%	0.666
*Marriage*					
Widowed	38	90.5%	37	90.2%	0.971
Married	4	9.5%	4	9.8%	0.971
Single	0	0.0%	0	0.0%	0
Divorced or others	0	0.0%	0	0.0%	0
*Occupation*					
Peasants	4	9.3%	5	11.6%	0.725
Employment	24	61.5%	23	60.5%	0.829
Unemployment	7	41.2%	5	26.3%	0.534
Retirement	1	2.0%	2	3.8%	0.557
Unknown	4	8.0%	5	9.8%	0.725
Others	3	6.5%	3	6.5%	1
*Comorbidities*					
Yes	2	4.7%	4	9.3%	0.397
No	41	95.3%	39	90.7%	0.397
*CRP difference*	35.42±39.00		34.74±43.86		0.950
*Neutrophil proportion difference*	74.32±16.16		75.39±9.40		0.730
*Neutrophil count difference*	6.07±3.09		5.56±2.00		0.410
*White blood cell count difference*	7.99±3.42		7.33±2.19		0.330

**Table 7:** Outcome comparison after PSM: Xiyanping combination versus penicillin.

Variable	Xiyanping combination		Penicillin		*P*
	N	%	N	%	
*Length of hospital stay*	8.33±3.51		8.69±3.56		0.384
*CRP difference*	44.20±53.48		-4.68±9.35		0.076
*Neutrophil proportion difference*	24.18±21.34		-2.57±6.87		0.027
*Neutrophil count difference*	4.03±3.26		-1.35±5.14		0.127
*White blood cell count difference*	3.27±2.66		18.22±40.37		0.513
*Treatment effect*					
Cure	21	15.1%	27	19.4%	0.341
Turn for the better	30	21.6%	36	25.9%	0.398
Not cured	3	2.2%	2	1.4%	0.652
Unknown	1	0.7%	0	0.0%	0.316
Death	80	57.6%	68	48.9%	0.149
Others	4	2.9%	6	4.3%	0.519

**Table 8:** Outcome comparison after PSM: Xiyanping combination versus cephalosporin.

Variable	Xiyanping combination		Cephalosporin		*P*
	N	%	N	%	
*Length of hospital stay*	7.84±3.31		8.47±3.29		0.070
*CRP difference*	40.86±46.81		14.24±61.12		0.108
*Neutrophil proportion difference*	20.40±18.60		6.82±11.64		0.006
*Neutrophil count difference*	3.85±5.96		1.11±5.31		0.112
*White blood cell count difference*	3.15±5.95		0.45±6.11		0.141
*Treatment effect*					
Cure	34	18.9%	36	20.0%	0.790
Turn for the better	59	32.8%	55	30.6%	0.650
Not cured	2	1.1%	1	0.6%	0.562
Unknown	2	1.1%	2	1.1%	1
Death	77	42.8%	78	43.3%	0.915
Others	6	3.3%	8	4.4%	0.586

**Table 9:** Outcome comparison after PSM: Xiyanping combination versus antiviral.

Variable	Xiyanping combination		Antiviral		*P*
	n	%	n	%	
*Length of hospital stay*	5.88±3.04		6.93±3.08		0.117
*CRP difference*	39.35±46.94		2.31±10.39		0.062
*Neutrophil proportion difference*	22.62±12.38		-0.49±14.44		0.003
*Neutrophil count difference*	3.00±2.73		-3.46±6.31		0.017
*White blood cell count difference*	2.10±2.69		-3.94±6.84		0.033
*Treatment effect*					
Cure	10	23.3%	15	34.9%	0.235
Turn for the better	27	62.8%	22	51.2%	0.276
Not cured	1	2.3%	1	2.3%	1
Unknown	0	0.0%	1	2.3%	0.314
Death	4	9.3%	2	4.7%	0.397
Others	1	2.3%	2	4.7%	0.557

**Table 10:** Cost comparison after PSM: Xiyanping combination versus penicillin.

Cost	Xiyanping combination		Penicillin		*P*
	Mean (CNY)	%	Mean (CNY)	%	
*Total hospitalization cost, (Mean ± SD)*	10,506.10±7,722.98		9,573.51±6,113.09		0.266
*Drug fees*	6242.54	59.4%	5466.81	57.1%	
TCM fees	1007.30	9.6%	921.24	9.6%	0.703
Western medicine fee	5235.25	49.8%	4545.57	47.5%	0.352
*Laboratory fee*	1287.65	12.3%	1105.44	11.5%	0.600
*Bed fee*	350.18	3.3%	338.33	3.5%	0.959
*Operation fee *	184.43	1.8%	272.19	2.8%	0.563
*Nursing fee *	93.83	0.9%	88.63	0.9%	0.929
*Inspection fee*	814.62	7.8%	907.12	9.5%	0.197
*Treatment fee*	807.64	7.7%	783.04	8.2%	0.967
*Another fee*	725.21	6.9%	611.94	6.4%	0.211

**Table 11:** Cost comparison after PSM: Xiyanping combination versus cephalosporin.

Cost	Xiyanping combination		Cephalosporin		*P*
	Mean (CNY)	%	Mean (CNY)	%	
*Total hospitalization cost, (Mean ± SD)*	8585.54±6325.91		8937.63±7638.90		0.634
*Drug fees*	4766.45	55.5%	4415.50	49.4%	
TCM fees	885.20	10.3%	676.27	7.6%	0.024
Western medicine fee	3881.25	45.2%	3739.23	41.8%	0.811
*Laboratory fee*	1381.49	16.1%	1441.17	16.1%	0.686
*Bed fee*	268.49	3.1%	319.03	3.6%	0.016
*Operation fee *	196.25	2.3%	553.60	6.2%	0.170
*Nursing fee *	65.89	0.8%	81.40	0.9%	0.125
*Inspection fee*	664.55	7.7%	873.40	9.8%	0.007
*Treatment fee*	666.38	7.8%	612.97	6.9%	0.694
*Another fee*	576.03	6.7%	640.55	7.2%	0.448

**Table 12:** Cost comparison after PSM: Xiyanping combination versus antiviral.

Cost	Xiyanping combination		Antiviral		*P*
	Mean (CNY)	%	Mean (CNY)	%	
*Total hospitalization cost, (Mean ± SD)*	3916.67±2084.50		8489.04±24390.60		0.227
*Drug fees*	1954.42	49.9%	3584.16	42.2%	
TCM fees	362.54	9.3%	195.83	2.3%	0.361
Western medicine fee	1591.88	40.6%	3388.33	39.9%	0.305
*Laboratory fee*	1281.74	32.7%	1892.40	22.3%	0.319
*Bed fee*	107.43	2.7%	173.31	2.0%	0.027
*Operation fee *	0.00	0.0%	617.48	7.3%	--
*Nursing fee *	24.90	0.6%	77.36	0.9%	0.162
*Inspection fee*	198.91	5.1%	410.69	4.8%	0.044
*Treatment fee*	156.35	4.0%	674.47	7.9%	0.245
*Another fee*	192.92	4.9%	1059.17	12.5%	0.035

**Table 13:** Results of CEA: Xiyanping combination versus penicillin.

	Cost (CNY)	Effecti-veness	C/E	Δ Incremental cost (CNY)	Δ Incremental effectiveness	ICER
*Hospitalization stay (day)*						
Xiyanping combination	10,506	8.33	1,261.22	932.00	-0.36	2,588.89
Penicillin	9,574	8.69	1,101.73			
*Cure rate (*%)						
Xiyanping combination	10,506	15.1	695.76	932.00	-4.30	
Penicillin	9,574	19.4	493.51			dominated

**Table 14:** Results of CEA: Xiyanping combination versus cephalosporin.

	Cost (CNY)	Effecti-veness	C/E	Δ Incremental cost (CNY)	Δ Incremental effectiveness	ICER
*Hospitalization stay (day)*						
Xiyanping combination	8,586	7.84	1,095.15	-352.00	-0.63	dominated
Cephalosporin	8,938	8.47	1,055.25			
*Cure rate (*%)						
Xiyanping combination	8,586	18.9	454.29	-352.00	-1.10	320
Cephalosporin	8,938	20	446.90			

**Table 15:** Results of CEA: Xiyanping combination versus antiviral.

	Cost (CNY)	Effecti-veness	C/E	Δ Incremental cost (CNY)	Δ Incremental effectiveness	ICER
*Hospitalization stay (day)*						
Xiyanping combination	3,917	5.88	666.16	-4,572.00	-1.05	dominated
Antiviral	8,489	6.93	1,224.96			
*Cure rate (*%)						
Xiyanping combination	3,917	23.3	112.23	-4,572.00	11.60	394.14
Antiviral	8,489	34.9	364.33			

## Data Availability

The data can be provided from corresponding authors upon reasonable request.
